# The chromatin remodelling component SMARCB1/INI1 influences the metastatic behavior of colorectal cancer through a gene signature mapping to chromosome 22

**DOI:** 10.1186/1479-5876-11-297

**Published:** 2013-11-28

**Authors:** Massimo Pancione, Andrea Remo, Caterina Zanella, Lina Sabatino, Arturo Di Blasi, Carmelo Laudanna, Laura Astati, Michele Rocco, Delfina Bifano, Paolo Piacentini, Laura Pavan, Alberto Purgato, Filippo Greco, Alberto Talamini, Andrea Bonetti, Michele Ceccarelli, Roberto Vendraminelli, Erminia Manfrin, Vittorio Colantuoni

**Affiliations:** 1 Department of Sciences and Technologies, University of Sannio, Via Port’Arsa, 11 82100 Benevento, Italy; 2 Department of Pathology, Surgery and Oncology “Mater Salutis” Hospital, ULSS21, Legnago, Verona, Italy; 3 Departments of Oncology and Pathology, Azienda Ospedaliera “G. Rummo”, Benevento, Italy; 4 Department of Anatomic Pathology, AORN Santobono Pausilipon, Naples, Italy; 5 Department of Pathology “G.B. Rossi” Hospital, University of Verona, Verona, Italy

**Keywords:** Integrase interactor 1, Colorectal cancer, Chromosome 22

## Abstract

**Background:**

INI1 (Integrase interactor 1), also known as SMARCB1, is the most studied subunit of chromatin remodelling complexes. Its role in colorectal tumorigenesis is not known.

**Methods:**

We examined SMARCB1/INI1 protein expression in 134 cases of colorectal cancer (CRC) and 60 matched normal mucosa by using tissue microarrays and western blot and categorized the results according to mismatch repair status (MMR), CpG island methylator phenotype, biomarkers of tumor differentiation CDX2, CK20, vimentin and p53. We validated results in two independent data sets and in cultured CRC cell lines.

**Results:**

Herein, we show that negative SMARCB1/INI1 expression (11% of CRCs) associates with loss of CDX2, poor differentiation, liver metastasis and shorter patients’ survival regardless of the MMR status or tumor stage. Unexpectedly, even CRCs displaying diffuse nuclear INI1 staining (33%) show an adverse prognosis and vimentin over-expression, in comparison with the low expressing group (56%). The negative association of SMARCB1/INI1-lack of expression with a metastatic behavior is enhanced by the *TP53* status. By interrogating global gene expression from two independent cohorts of 226 and 146 patients, we confirm the prognostic results and identify a gene signature characterized by SMARCB1/INI1 deregulation. Notably, the top genes of the signature (*BCR, COMT, MIF)* map on the long arm of chromosome 22 and are closely associated with SMARCB1/INI1.

**Conclusion:**

Our findings suggest that *SMARCB1/INI1*-dysregulation and genetic hot-spots on the long arm of chromosome 22 might play an important role in the CRC metastatic behavior and be clinically relevant as novel biomarkers.

## Background

The chromatin remodelling (CR) complexes dynamically regulate transcription by using the energy from ATP hydrolysis to reposition nucleosomes and modulate accessibility of specific genes to the transcriptional machinery [1,2]. Recently, inactivating mutations in the CR complexes have been identified at high frequency in a variety of tumors, highlighting the widespread role of epigenome alterations in tumor suppression or oncogenic activation [1]. Integrase interactor 1 (INI1, also known as SMARCB1) is a core subunit of the SWI/SNF ATP-dependent CR complex encoded by the corresponding gene at chromosomal position 22q11.2 [3-5]. SMARCB1/INI1 is ubiquitously expressed in normal cells and can be readily identified by immunohistochemistry. *SMARCB1/INI1* germ-line mutations were first described in the malignant rhabdoid tumors (MRT) of infancy and atypical theratoid/rhabdoid tumors of the central nervous system and define a hereditary condition known as “Rhabdoid predisposition syndrome” [3-6]. Deletions at chromosome 22 or loss of *SMARCB1/INI1* expression have also been implicated in the pathogenesis of additional tumor types: renal medullary carcinomas, epithelioid sarcomas, myoepithelial carcinomas and extraskeletal myxoid chondrosarcomas [7]. Although SMARCB1/INI1 is the most extensively studied subunit of the SWI/SNF complex, very little is known about its role in the pathogenesis of colorectal cancer (CRC) [8]. Recently, we reported that *SMARCB1/INI1* inactivation or, alternatively, a genomic rearrangement at the chromosome region 22q12 are involved in Rhabdoid Colorectal Tumor (RCT), a rare and highly aggressive neoplasm of the gastrointestinal tract [9,10]. *SMARCB1/INI1*-deficient mice develop rapidly aggressive undifferentiated sarcomas, implying a cancer-related function [11]. Notably, in the same mouse model, the conditional inactivation of *TP53* leads to a dramatic acceleration of tumor formation and a wider spectrum of cancers than those seen in *TP53* deficient mice alone [12]. These results suggest a cooperative effect of both genes to prevent oncogenic transformation and a dominant role of *SMARCB1/INI1* to hamper cancer aggressiveness. Despite the evidence in mouse models, the link between *SMARCB1/INI1* alterations and the molecular changes underlying CRC progression remains still poorly understood. In order to shed light on the biological role of *SMARCB1/INI1*, in this study we investigated its expression profile and evaluated the relationship between molecular alterations and clinico-histological markers of dedifferentiated and aggressive colorectal carcinomas. We hypothesize that its assessment might be clinically relevant to predict CRC prognosis.

## Materials and methods

### Tumor samples and TMA construction

Colorectal cancer specimens and matched normal mucosa were collected at two institutions, Fatebenefratelli Hospital, Benevento, and Legnago Hospital, Verona, Italy. This study was carried out according to the principles of the Declaration of Helsinki with appropriate patient’s informed consent and approved by the Institutional Review Board of both hospitals. Altogether, a total of 134 patients, 85 men and 49 women with mean age of 70.5 ± 11.8 were analyzed. The tumors were classified and graded according to the criteria of the TNM and tumor stages I-IV classification systems, (Table 1). None of the patients had a familial history of intestinal dysfunction or CRC, had received chemotherapy or radiation prior to resection nor had taken non-steroidal anti-inflammatory drugs on a regular basis. For each patient, the date of colon cancer diagnosis, date of last follow-up, and vital status at last follow-up (*i.e.,* living or deceased) were recorded. TMAs were constructed from archival tissue blocks of normal and colorectal cancer using a Beecher tissue microarray instrument (Beecher Instruments, Hacken-sack, NJ, USA). Tissue cylinders, with a diameter of 0.6 mm, were punched from paraffin blocks in demarcated areas on parallel haematoxylin&eosin-stained sections. Three separate cores were sampled from each block deposited into a recipient master paraffin block. Each core was placed 1 mm apart on the x-axis and 1.5 mm apart on the y-axis of the master block. In total, 12 microarrays paraffin block were prepared, 4 μ thick sections were cut from each TMA block and stained with haematoxylin&eosin. Microarray sections were then reviewed to ensure that the sections from each case were morphologically similar to those of the corresponding whole tissue section and represented cancerous or normal epithelial cells. Further 4 μ thick sections were then cut from each of the master blocks for immunohistochemical (IHC) analyses, the cores containing too little tumor sample were not included in the study. Due to technical problems and/or tissue exhaustion, the number of lesions that were available for evaluation by immunohistochemistry included 134 carcinomas (Table 1).

**Table 1:** **Correlation between** SMARCB1/INI1 **expression pattern and patients’ clinico-pathological parameters**

**Parameters**		**n**	**INI1**			***P *** **value**
			**Neg (%)**	**Low (%)**	**High (%)**	
**Age**	≤60	22	1 (4.5)	12 (54.5)	9 (41)	0.456
	>60	112	14 (12.5)	63 (56.2)	35 (31.3)	
**Sex**	F	49	9 (18.4)	21 (42.6)	19 (39)	0.056
	M	85	6 (7.1)	54 (63.5)	25 (29.4)	
**Location**	Proximal	51	5 (9.8)	34 (66.6)	12 (23.6)	0.136
	Distal	83	10 (12)	41 (49.4)	32 (38.6)	
**Histology**	ADC	108	10 (9.2)	62 (57.4)	36 (33.4)	0.667
	A-Muc	17	3 (17.6)	9 (52.9)	5 (29.5)	
	Other	9	2 (22.2)	4 (44.4)	3 (33.3)	
**Grade**	Well/mod	109	7 (6.4)	67 (61.5)	35 (32.1)	0.003*
	Poor	25	8 (32)	8 (32)	9 (36)	
**N stage**	N0	88	7 (7.9)	51 (62.5)	30 (29.6)	0.387
	N1	24	3 (12.5)	14 (58.3)	7 (29.2)	
	N2	22	5 (22.7)	10 (45.4)	7 (31.9)	
**LiverMet**	Negative	93	4 (4.3)	58 (62.4)	31 (33.3)	0.001*
	Positive	41	11 (26.8)	17 (41.4)	13 (31.8)	
**Stage**	I	12	0	5 (41.6)	7 (58.4)	0.002*
	II	63	3 (4.8)	42 (66.6)	18 (28.6)	
	III	20	1 (5)	12 (60)	7 (35)	
	IV	39	11 (28.2)	16 (41)	12 (30.8)	
**Total**		134	15 (11)	75 (56)	44 (33)	

Tumor classification was based on the TNM (Tumor-Node-Metastasis) system, according to the criteria of the International Union Against Cancer (UICC).

*Abbreviations:Proximal* caecum, ascending and transverse colon, *Distal* descending and sigmoid colon, rectum, *Adc* adenocarcinoma, *AD-Muc* adenocarcinoma with a mucinous component below 50%, *Other* squamous or rhabdoid, *Well/mod* well and moderately differentiated, *Poor* poorly differentiated adenocarcinoma, *Liver Met* Liver metastasis.*Chi-square statistic significant at the 0.01 level.

### Immunohistochemistry

The TMAs were serially sectioned at 4 μ, dewaxed in xylene and rehydrated through graded alcohol to water. Slides were subjected to microwave antigen retrieval in 10 mM Citrate buffer (pH of 6.0) before incubation with the primary antibodies. The following antibodies, at 1:100 dilution, were employed: SMARCB1/INI1 clone 25/BAF47; (DAKO Cytomation, Glostrup, Denmark). CK20 clone Ks 20.8; vimentin clone VIM 3B4; p53 clone Bp53-11; (Novocastra Laboratories, Newcastle, UK); CDX2 clone EPR2764Y (Ventana Medical Systems, Tucson, AZ, USA). Automated immunohistochemistry system (Ventana Medical Systems, Tucson, AZ, USA) was employed to detect immunostaining as previously reported [10,13]. Finally, the sections were counterstained with hematoxylin, dehydrated, and cover-slipped. In each run, primary antibodies were omitted in negative controls.

### Evaluation of immunohistochemistry

All immunohistochemical results were interpreted by 2 independent observers (A. R, and M. P.) blinded to clinical data and laboratory results. For SMARCB1/INI1, p53 and vimentin the immunostaining was recorded regardless of intensity, according to the proportion of positive neoplastic cells. According to the number of positive tumor cells, we stratified the carcinomas into three groups: 1) “Low expression”, in which the positivity was observed in a limited number of tumor cells, scattered in a background of either negative or weakly positive tumor cells; this subgroup was also defined as Partly positive; 2) “High expression” or strongly diffuse expression, corresponding to an homogeneous staining in virtually all tumor cells 3) “Negative expression” when less than 5% of tumor cells were positive. Positivity in normal colonic mucosa, inflammatory and stromal cells adjacent to neoplastic cells served as positive internal controls. For CK20 and CDX2, the staining in less than 5% of tumor cells was scored as negative. For each marker, normal colonic mucosal tissue was used as positive control.

### Mismatch repair, MSI and CIMP analysis

To evaluate mismatch repair, the following antibodies at a dilution 1:100, were used: anti–MLH-1 clone (M1); anti-MSH-2 clone (G219-1129); anti-MSH6 clone 44; anti-PMS2 clone EPR394; (Ventana Medical Systems, Tucson, AZ, USA). The tumors were defined as mismatch repair-deficient when they showed an absence of nuclear staining in at least one of following marker: MLH1 or MSH2 or MSH6 or PMS2. Inflammatory and stromal cells adjacent to neoplastic cells served as positive internal controls. Microsatellite instability (MSI) assessment in both mismatch repair-deficient or proficient cases was performed comparing tumor DNA and matched normal mucosa through a panel of highly-specific five mononucleotide repeats, as described [14]. An agreement of the 95% between MSI and MMR status was obtained, supporting the use of MMR profile for subsequent analyses. Genomic DNA isolation and sodium bisulphite modification were carried out as reported. The converted DNA was subjected to quantitative methylation specific PCR as reported [10,13]. The following genes (*RUNX3, IGF2, SOCS1, NEUROG1, CDKN2A* (p16) and h*MLH1*) with methylation levels greater than 15% were considered positive. Tumors with at least three methylated loci were classified as CpG island methylator phenotype (CIMP)-positive and the remaining cases as CIMP-negative [10,13]. The primers for promoter methylation analysis have already been reported.

### Cell culture, migration, western blot and qRT-PCR analysis

Human CRC derived cell lines DLD1, HCT116, LoVo, RKO and SW480 were purchased from ATCC and cultured as recommended. Cell migration was evaluated by the wound-healing as previously described [10] and ref. therein. Western blot analysis and qRT-PCR were performed as already reported [10] and ref. therein. Expression levels were normalized to β-actin or to GAPDH mRNA, respectively. A detailed description of the primer sets will be provided upon request.

### Independent CRC data sets and statistical analysis

The following independent, publically available CRC datasets, deposited in the Gene Expression Omnibus (GEO) GSE17536, GSE17537 and GSE41258 series (http://www.ncbi.nlm.nih.gov/geo) were analyzed to validate *SMARCB1/INI1* expression and its prognostic significance [15,16]*.* The GSE17536 and GSE17537 pooled series (cohort I) consists of 226 patients; while the GSE41258 series (cohort II) consists of 146 patients [15,16]. Disease-specific survival was considered as a prognostic variable, whereas, the data on *TP53* mutations status were available only for cohort II. A fold-change of at least 1.5 (*p* value <0.05) was used to identify up- and down-regulated genes, respectively. Volcano plot analysis was employed to visualize differential expression. In order to find differentially expressed genes (DEGs) co-regulated with *SMARCB1/INI1,* a heat map with hierarchical clustering analysis was performed. The DEGs were separated in two clusters using a random-variance t test. Subsequently, they were selected for Gene Ontology (GO) terms and pathway analysis. Ingenuity Pathways Analysis (IPA; Ingenuity Systems, http://www.ingenuity.com) was used for gene set enrichment analysis and gene network analysis. Statistical analyses were performed using GeneSpring R/bioconductor v.12.5. Data are reported as median or mean and standard deviation (SD), and the mean values compared using the Student’s t test, as indicated. The χ^2^ or Spearman tests were employed to assess the association of markers and clinico-pathological parameters. Univariate analyses were performed by using Kaplan-Meier estimates and log-rank tests, with raw score data obtained for each individual biomarker. A Cox regression model stepwise selection procedure for was used to identify those markers that independently predict disease outcome whereby hazard ratios (HR), 95% confidence interval (95% CI) and significance levels were estimated. Statistical analyses were carried out with the SPSS (version 15.0) for Windows (SPSS Inc., Chicago, Ill., USA). Results were considered statistically significant when a *p ≤* 0.05 was obtained.

## Results

### SMARCB1/INI1 expression profile in colorectal cancer and matched normal mucosa

**Figure 1:**
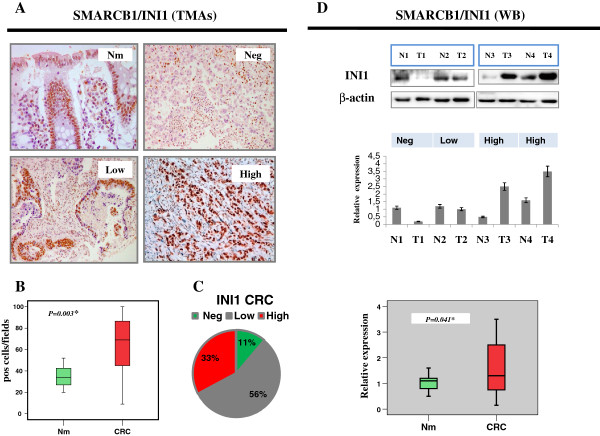
**SMARCB1/INI1 expression analysis in Tissue Microarray of CRC and matched normal mucosa. (A)** Examples of TMA cores representative of the normal mucosa and CRC specimens stained with SMARCB1/INI1; the immunostaining pattern allows to divide tumors into three categories, Neg, Low, High**. (B)** The box-plot shows the SMARCB1/INI1score (number of positive cells in 10-high power fields) in paired normal mucosa and tumor samples (60 cases). **(C)** SMARCB1/INI1 expression pattern expressed as percentage of cases for each category in all 134 CRCs. **(D)** Four representative frozen CRC specimens (T) and matched normal mucosa (N) from the same cohort of patients are analyzed by immunoblot. The β-actin is used as loading control to normalize SMARCB1/INI1 band intensities. The histogram reports quantitative expression levels of SMARCB1/INI1 after normalization. The box-plot shows that SMARCB1/INI1 expression detected by western blot in normal mucosa is lower than tumor samples (20 cases). The *p* value is reported in each graph.

In the normal mucosa, SMARCB1/INI1 nuclear positivity was evenly distributed between proliferative and differentiated colonic cells (Figure 1A). In few cases (5/60, 8%), we observed a stronger positivity in the proliferative compartment of the crypts. To identify cancer-specific alterations, we first investigated the differences in SMARCB1/INI1 expression in CRCs and paired normal mucosa (Figure 1A). CRC samples exhibited a higher percentage of SMARCB1/INI1-positive cells than matched normal colonic mucosa (Figure 1B). We further analyzed CRC samples and classified SMARCB1/INI1 expression pattern into three groups, according to the proportion and distribution of positive neoplastic cells. By applying this criterion, we detected a moderate expression in 56% (75/134) of tumors, classified as Low or Partly positive; 33% (44/134) had a diffuse and stronger positivity and classified as High, while 11% (15/134) did not show any significant SMARCB1/INI1 immunoreactivity, classified as Negative (Figure 1C). The expression profile was validated on twenty representative cases (5 negative and 15 positive), by evaluating the consensus between each core of the TMAs and the corresponding whole tissue section. We found no discordance, supporting the value of the TMA method to screen SMARCB1/INI1 expression in our CRC dataset. To further corroborate the IHC expression profile and have more quantitative data, twenty selected frozen CRC specimens and matched normal mucosas from the same cohort of patients were analyzed by western blot. The bands were quantitated by densitometry after normalization to β-actin for protein loading. SMARCB1/INI1 showed variable expression levels in CRC specimens as compared to case-matched normal tissue. Five tumors, defined SMARCB1/INI1-negative, showed lack of SMARCB1/INI1 protein as compared to normal mucosa, confirming the IHC results (Figure 1D). In contrast, five tumors were SMARCB1/INI1-positive as the expression was significantly higher than controls. The remaining ten cases showed no significant changes versus the normal mucosa. Although the data referred only to twenty cases, they confirmed the specificity of the results and reinforced the differences between normal and tumor samples detected by IHC on TMAs (Figure 1D).

### SMARCB1/INI1 expression profiles correlate with poorly differentiated tumors and liver metastasis

**Figure 2:**
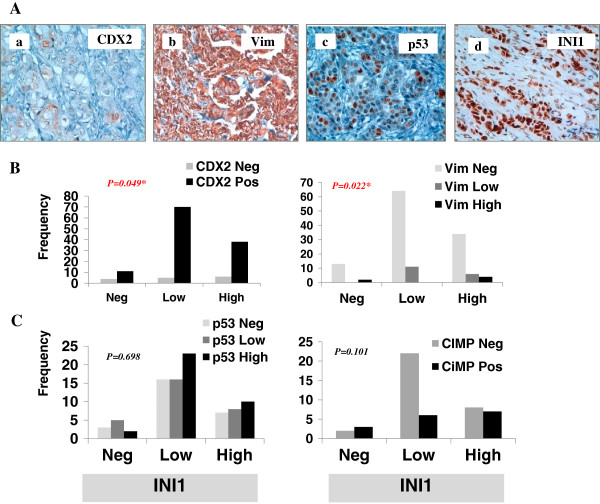
**Correlation between SMARCB1/INI1 expression and different molecular markers. (A)** CDX2, vimentin, p53 and SMARCB1/INI1 immunostaining profile in a poorly differentiated tumor **(a-d). (B)** The SMARCB1/INI1 expression profile is divided into three categories Neg, Low and High and correlated with CDX2 and vimentin expression pattern in tumor cells. **(C)** The same analysis takes into account p53 expression and the CpG island methylator phenotype (CIMP) status. Tumors with at least three methylated markers (*RUNX3, IGF2, SOCS1, NEUROG1, CDKN2A* and h*MLH1*) were classified as CIMP-positive, the remaining as CIMP-negative. The *p* value is reported in each graph.

We then associated the SMARCB1/INI1 expression patterns with patients’ clinico-pathological features, immunohistochemical and molecular markers of tumor differentiation. SMARCB1/INI1-negative immunostaining showed a significant relation with poor differentiation, presence of liver metastasis and advanced tumor stage IV (Table 1). No statistically significant difference was found taking into account other clinico-pathological features such as: age, gender, histology, tumor location and lymph node metastasis (Table 1). Next, we examined whether its expression could correlate with multiple biomarkers such as: CDX2, CK20, vimentin, p53, CIMP and MMR status (Figure 2A, B and C and data not shown). SMARCB1/INI1-negative tumors showed lower CDX2 positivity than any other group (*p =* 0.049). The same group exhibited a diffuse pattern of vimentin overexpression. Unexpectedly, also SMARCB1/INI1-high tumors were markedly vimentin positive (Figure 2B). We did not detect any significant correlation with either p53 expression or CIMP-positive tumors (Figure 2C).

We sought to investigate the relationship of each of the markers analyzed with the MMR status, by dividing the CRCs in two groups according to the proficient or deficient condition. 23% (23/134) of the cases were MMR deficient (MMR negative); as expected, they occurred more frequently at the right colon and were poorly differentiated tumors. Consistent with previous studies, this subgroup exhibited lack of CDX2, CK20 and p53 expression (Additional file 1: Table S1) and, interestingly, higher vimentin positivity than the MMR proficient CRCs that showed instead low vimentin levels (94 vs 6%). Finally, we detected no correlation between the different SMARCB1/INI1 expression profiles and the MMR or MSI status (Additional file 1: Table S1, data not shown). These results indicate that loss of SMARCB1/INI1 expression is associated with poorly differentiated tumors and presence of liver metastasis. Furthermore, even a significant proportion of CRCs with high SMARCB1/INI1 expression exhibit a marked vimentin positivity.

### Altered SMARCB1/INI1 expression correlates with patients’ prognosis in our CRC dataset

**Figure 3:**
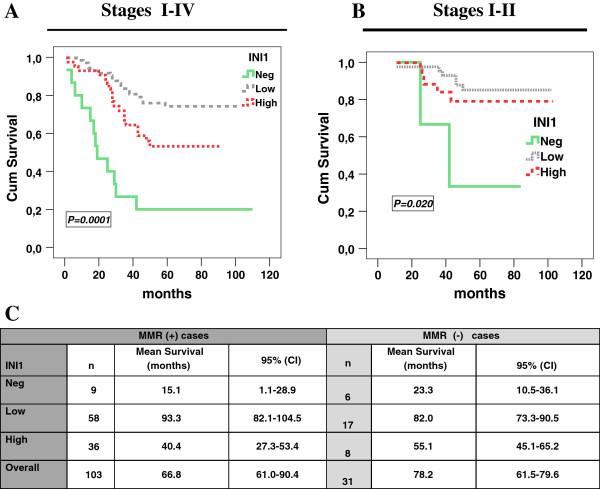
**SMARCB1/INI1 expression in CRC and its impact on patients’ survival. (A)** Overall survival (OS) referred to all tumor stages (I-IV) is estimated using the Kaplan–Meier method and stratifying the patients according to three categories of SMARCB1/INI1 expression (Neg, Low, High). **(B)** The same Kaplan-Meier survival analysis is carried out taking into account only tumor stages I-II. **(C)** Mean survival time referred to the three categories of SMARCB1/INI1 expression and stratified according to the mismatch repair (MMR) status. MMR (+) and MMR (−) indicate MMR proficient and deficient tumors, respectively. *p <0.01.* The *p* value is reported in each graph.

**Figure 4:**
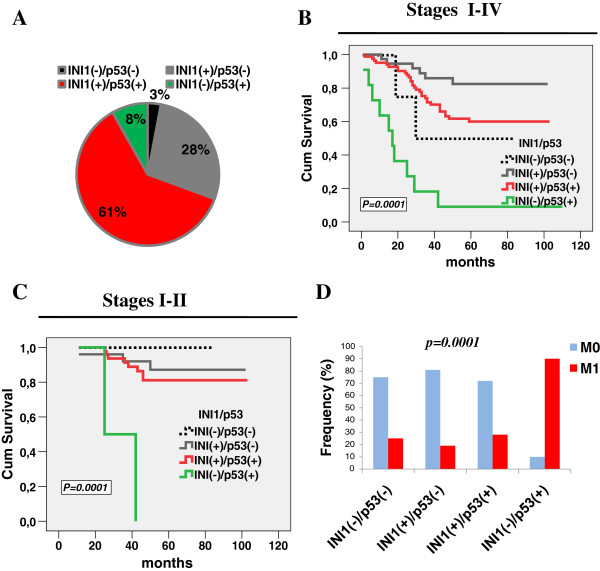
**SMARCB1/INI1 and p53 expression impacts aggressive features of tumors. (A)** Frequency of the four CRC categories according to the various combinations of p53 and SMARCB1/INI1 expression. **(B)** OS analysis for each category referred to all tumor stages (I-IV) **(C)** The same analysis is carried out taking into account only tumor stages I-II. **(D)** Frequency of liver metastasis according to the various combinations of p53/ SMARCB1/INI1 expression. M0 and M1 indicate absence or presence of liver metastases, respectively. The *p* value is reported in each graph.

In our cohort, cancer related death occurred in 35.8% of the cases (48/134 patients). We stratified patients’ overall survival (OS) into three categories according to the SMARCB1/INI1 expression patterns. Low SMARCB1/INI1-expressing tumors had the best prognosis as compared to those with High or Negative expression (Figure 3A). The SMARCB1/INI1-negative subgroup preserved the worst impact on patients’ survival also in tumor stages I-II or when adjusted for all tumor stages in a multivariate analysis (Figure 3B and data not shown). To investigate whether the prognostic impact of SMARCB1/INI1 was dependent upon the MMR, we stratified the tumors according to the MMR deficient or proficient status. Two groups of patients, the SMARCB1/INI1-negative or -high expressing ones, were associated with a shorter survival time than the low expressing ones in both MMR-proficient and deficient CRCs (Figure 3C). A multivariate model showed that SMARCB1/INI1 expression preserves a prognostic significance when adjusted for MMR status (data not shown). Since SMARCB1/INI1 and p53 co-inactivation can accelerate the rate of tumorigenesis, we explored the effects of such a combination on patients’ outcome. To this end, we classified tumors into 4 groups according to positive or negative expression (Figure 4A). Interestingly, the SMARCB1/INI1^-^/p53^+^ group (8% of cases, 11/134 patients) showed a very short OS in all tumor stages I-IV or stages I-II alone, when compared to any other SMARCB1/INI1/p53 combination (Figure 4B,C). In agreement with these data, almost the entire SMARCB1/INI^-^/p53^+^ subgroup (90%) developed liver metastases with respect to any other group (Figure 4D). To further support our findings, we interrogated a CRC independent dataset, validation cohort II, from which the transcriptional profile of *SMARCB1/INI1*, *TP53* mutation status and clinical follow-up are publicly available [16]. We confirmed that the *SMARCB1/INI1* down-regulation, combined with *TP53* mutations, correlated with a poorer patients’ prognosis than any other group (Additional file 1: Figure S1A). Altogether, these results indicate that SMARCB1/INI1-negative or -high expression is associated with an adverse CRC prognosis regardless of the MMR status and is influenced at least in part by the *TP53* status.

### *SMARCB1/INI1* expression is validated in two independent cohorts of patients and reveals a gene signature mapping to chromosome 22

**Figure 5:**
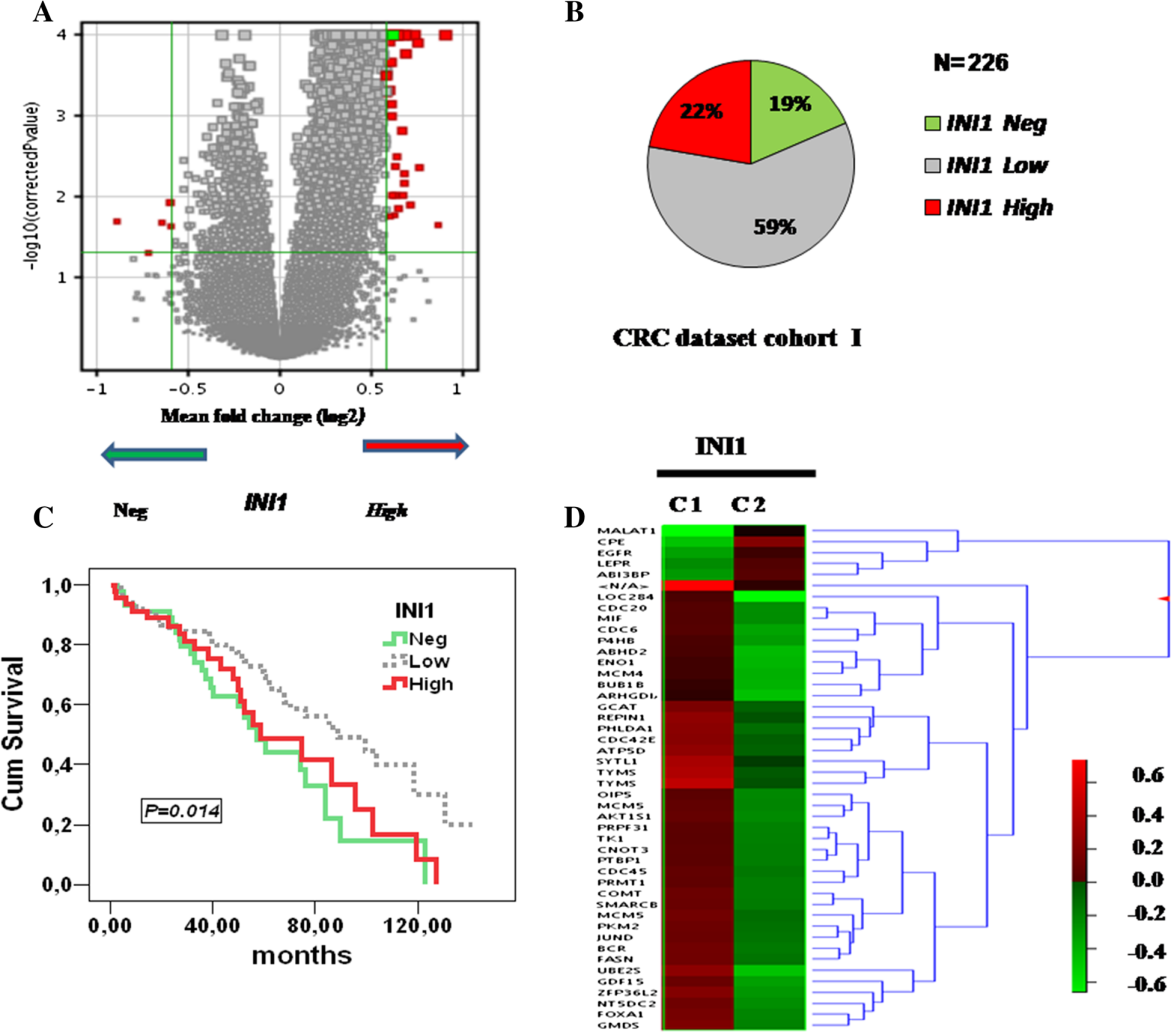
**SMARCB1/INI1 expression profile is validated in an independent CRC microarray dataset, cohort I. (A)** Volcano plot shows the graphical breakdown of the statistical analysis of SMARCB1/INI1 microarray data; The (x-axis) is the base 2 logarithm of the fold change, and the (Y-axis) is the negative base 2 logarithm of the q value (or adjusted *p*-value). Thresholds for both the statistical (q < 0.05) and the biological significance are highlighted and assembled in the top left and top right corner of the graph. The fold-changes with significant *p* values corresponding to SMARCB1/INI1-Neg and SMARCB1/INI1-High tumors (on the vertical axis) show that a 1.5-fold up- or down-regulation in gene expression is equivalent to log-ratios of +0.5 and −0.5; **(B)** Frequency of the identified subgroups displaying differential *SMARCB1/INI1* transcription in the validation series, cohort I, expressed in percentage; **(C)** Kaplan-Meier survival analysis is carried out taking into account each category; **(D)** Heat-map of differentially expressed genes. A hierarchical clustering method was used to construct the gene tree as described in Materials and Methods. The lists of differentially expressed genes with a t-test *p-*value *<0.05* including multiple testing corrections were generated (for details, see also Additional file 1: Table S1). Data are shown in a matrix format: each row represents a single gene and each column represents a group. Red indicates overexpressed genes (expression levels over the median) and green indicates underexpressed genes (expression levels below the median; see legend). The pattern and length of the branches in the dendrograms reflect the relatedness of the samples or the genes. The *p* value is reported in each graph.

We further validated the *SMARCB1/INI1* expression profiles and its association with patients’ outcome by interrogating two CRC independent datasets, validation cohorts I and II, respectively [15,16]. We computed the differences in gene expression by applying a fold-change of at least 1.5, and divided the microarray data into three quartiles (Figure 5A). In validation cohort I, 42 out of 226 patients (19%) were included in the 1st quartile and classified as *SMARCB1/INI1*-Negative; 132 (59%), in the 2nd and 3rd quartiles and classified as Low; 50 (22%) in the 4th quartile and classified as High (Figure 5B). Notably, these expression profiles were comparable with those observed in our CRC cohort, suggesting that *SMARCB1/INI1* expression at mRNA and protein level is stably maintained also in an independent dataset.

We next examined the association of *SMARCB1/INI1* expression with disease specific survival for the 226 patients of validation cohort I, whose follow-up data were available. Disease specific survival referred to the three categories showed that *SMARCB1/INI1-*Negative- or -High patients had a shorter survival time than Low expressing ones (Figure 5C). Remarkably, similar results were obtained taking into account cohort II, an independent series of 146 patients (Additional file 1: Figure S1B). On the basis of these observations, we focused on two main groups, *SMARCB1/INI1*-Negative (down-regulated) and *SMARCB1/INI1-*High (up-regulated) tumors that significantly correlated with patients’ survival. The separation in two clusters was further confirmed by generating a two-dimensional hierarchical clustering heatmap. By this approach, we identified a robust set of genes, about 50, which significantly discriminated between *SMARCB1/INI1*-up and -down regulated tumors (Figure 5D and Additional file 1: Table S2). Overall, the differentially expressed genes were significantly enriched in GO biological processes including: gastrointestinal cancer, cell cycle control, chromosomal replication and epithelial cell differentiation (Additional file 1: Figure S2A, B).

**Figure 6:**
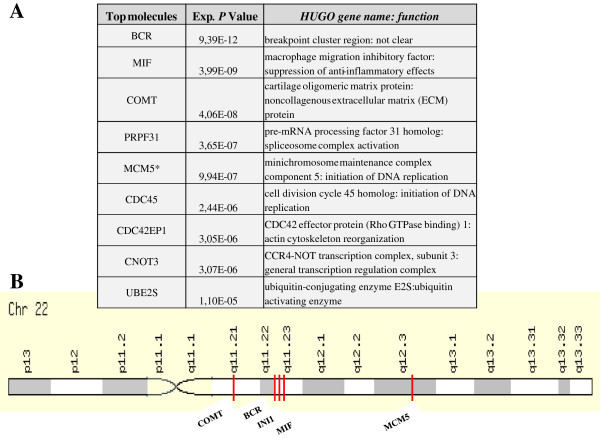
**The top ranked genes, identified in Cohort I, map to the long arm of chromosome 22 and are closely linked to*SMARCB1/INI1.*(A)** Top genes and biological functions that varied most in terms of differential expression across tumor samples displaying SMARCB1/INI1 down (Neg) or up-regulation (High). **(B)** The top deregulated genes (*BCR, MIF, COMT* and *MCM5*) with the highest statistically significant *p* values are located on the long (q) arm of chromosome 22 close to *SMARCB1/INI1*.

Most notably, a cluster of loci, which represents the top differentially expressed genes (*BCR, COMT, MCM5* and *MIF*) mapped to the same long (q) arm of chromosome 22 where *SMARCB1/INI1* resides (Figure 6A,B). In particular, two of the most coregulated genes (*BCR* and *MIF*) were located on the same cytogenetic band 22q11.23, about 60 kb apart from *SMARCB1/INI1* (Figure 6A,B). The results obtained from cohort I were confirmed by interrogating cohort II. Also in this case, the top differentially expressed genes were localized close to *SMARCB1/INI1,* expanding the list of deregulated genes that are mapped to chromosome 22 (Additional file 1: Figure S1C).

Finally, to verify whether the changes in *SMARCB1/INI1* expression were associated with variations of the selected genes, we investigated a panel of four representative human CRC cell lines (Additional file 1: Figure S3A-D). Interestingly, in poorly differentiated and more invasive RKO and DLD1 cell lines, we did confirm that the top deregulated genes were significantly associated with molecular features of enhanced vimentin and reduced CDX2 expression (Additional file 1: Figure S3C-D). The association between co-regulation and gene co-localization was confirmed by performing interphase FISH at 22q12 locus in a subgroup of CRC specimens (data not shown).

## Discussion

The chromatin remodelling complexes mobilize nucleosomes to expose DNA to the transcriptional machinery. Alterations of these complexes are emerging as a critical step in carcinogenesis; in fact, high-frequency mutations in SWI/SNF members have been found in a variety of cancers by whole genome sequencing [2]. SMARCB1/INI1 is a core subunit of the SWI/SNF complex and a recognized hallmark for the diagnosis of MRT and other mesenchymal cancers [4,7]. Negative SMARCB1/INI1 expression is quite rare in epithelial tumors and none of the studies published so far has addressed its role in colorectal cancer [7,17]. Only few SWI/SNF components (*BRM, BRG* and *ARID1A*) have been reported mutated or deregulated in colon cancer; limited functional insights into the mechanisms of oncogenesis promoted by chromatin remodelling complexes are available so far. Even more, the prognostic significance of a large number of SWI/SNF subunits remains unknown [17-21]. Recently, we found that SMARCB1/INI1 expression was either negative or high in rhabdoid colorectal tumors and in a small group of sporadic CRCs [9,10].

In the present study, we investigated the SMARCB1/INI1 expression profiles in a larger CRC series and found that the majority (89%) express SMARCB1/INI1 with two distinct patterns of nuclear positivity, low (56%) and high (33%), respectively. The SMARCB1/INI1 nuclear positivity observed in the low expressing group resembled that detected in 60 normal colon tissues. A small group that accounts for 11% of our CRC series displayed a negative SMARCB1/INI1 immunostaining. Notably, negative SMARCB1/INI1 expression was related to poorly differentiated tumors and high frequency of liver metastases disclosing an association between its altered expression and the CRC subgroups more prone to metastatic spreading. SMARCB1/INI1 negative tumors frequently showed loss of CDX2 and high expression of vimentin, two key markers involved in colonic differentiation and mesenchymal phenotype, respectively. Unexpectedly, enhanced vimentin positivity was also found in the group displaying diffuse SMARCB1/INI1 expression.

*SMARCB1/INI1* loss-of-function mutations or haploinsufficiency are recurrent in a variety of tumors, especially with rhabdoid features [4,7,17]. The molecular mechanisms underlying SMARCB1/INI1 protein inactivation in CRC were not explored in the present study; however, in agreement with recent data, we ruled out hypermethylation of the *SMARCB1/INI1* promoter region in our CRC cohort (our unpublished data) [17,21,22]. A recent comprehensive genome-wide analysis on 276 CRCs has identified *SMARCB1/INI1* mutations in less than 1% of cases [21]. These results suggest that epigenetic events might be responsible for *SMARCB1/INI1* inactivation because mutations alone do not fully explain the frequent variations in expression detected in CRCs. Further investigations are needed to answer this question.

The morphological revision of the slides from the 15 SMARCB1/INI1-negative tumors (7%, 1/15) revealed that only one showed a composite rhabdoid histology. The patient had a very short survival time (1 month), confirming the aggressive nature of this subgroup [8-10,13]. Unlike others RCTs, we found a *KRAS* mutation, no *BRAF* mutations nor microsatellite instability. These findings reinforce our previous data, implying that *SMARCB1/INI1* plays a crucial role in later stages of colon carcinogenesis [4,9,10].

The most striking finding of our study is the association between loss of *SMARCB1/INI1* expression and a worse clinical outcome, regardless of the tumor stage and MMR status. Unexpectedly, even SMARCB1/INI1-high expression is an adverse prognostic indicator in comparison with SMARCB1/INI1-low expressing tumors. The reasons for this apparent contradiction are not clear: they might be linked to the specific deregulated cross-talks between chromatin remodelling components, acquisition of mesenchymal markers and genomic alterations such as chromosomal instability (CIN). Although still debated, it has been suggested that *SMARCB1/INI1* could have a critical function in determining aneuploidy [23]. Indeed, a subgroup of CRCs with enhanced SMARCB1/INI1 expression has a consistent proportion of aneuploid cells, even exhibiting MMR deficiency (our unpublished data); these latter tumors, in fact, typically show a near-diploid karyotype [8]. Whether and how SMARCB1/INI1 dysfunctions are causally implicated in genomic instability remains controversial. We further investigated the SMARCB1/INI1 prognostic significance by exploring its effect in combination with the *TP53* status. Interestingly, the SMARCB1/INI1^-^/p53^+^ tumor group is closely correlated with very short survival and liver metastases when compared with other SMARCB1/INI1/p53 combinations, demonstrating a cooperative effect of both genes in restraining cancer aggressiveness in CRC advanced stages [12]. These results evoke the dramatic increase in tumor formation and metastasis obtained by inactivating *TP53* in *SMARCB1/INI1*-heterozygous mice. The clinical relevance of deregulated *SMARCB1/INI1* expression is confirmed in two independent CRC datasets of 226 and 146 patients, respectively, providing support to our findings. By interrogating genome-wide expression data, we identified several genes that were coordinately down- or up-regulated and separated in two distinct clusters. Notably, the top genes of the signature (*BCR, COMT, MIF)* map to the long arm of chromosome 22 at the cytogenetic band 22q11.23, closely associated with SMARCB1/INI1. The gene expression signature was confirmed also in CRC cell lines displaying molecular features of enhanced vimentin expression, reduced CDX2 and more mesenchymal phenotype. A chromosomal rearrangement (translocation/deletion) at 22q12 has recently been identified in a RCT and correlated with high SMARCB1/INI1 expression [9,10]. A further translocation involving TTC28 at 22q12.1 or focal amplification of multiple genes mapped at 22q12.3 has been reported by the Cancer Genome Atlas Network and correlated with tumor aggressiveness [21]. Based on these evidences, is tempting to speculate that a number of alterations, such as translocations or amplifications, involving a specific region on the long arm of chromosome 22 might be associated with clinical aggressiveness and a more mesenchymal phenotype.

In conclusion, we demonstrate that SMARCB1/INI1 deficiency, alone or in combination with *TP53* mutations, influences the CRC aggressive behavior, regardless of the MMR status. Surprisingly, even SMARCB1/INI1 diffuse expression is associated with poor survival, as confirmed in two independent cohorts of patients. We identify several over-expressed or repressed genes located on chromosome 22, close to *SMARCB1/INI1* and coordinately deregulated. Our findings suggest that *SMARCB1/INI1* and genetic hot spots mapping to the long arm of chromosome 22 play an important role in tumor metastatic spreading. SMARCB1/INI1 might then be a useful clinical prognostic marker to complement the histological examination and grading and to select patients for adjuvant medical treatments. Mechanistic and larger clinical studies are needed to define how chromatin remodelling components and which specific genomic rearrangements influence the CRC metastatic behavior.

## Competing interests

The authors declare that they have no competing interests.

## Authors’ contributions

Conceived the ideas for this study MP, AR; pathology analysis AR, EM, ADB; MR, DB; Acquisition, analysis and interpretation of data (acquired and managed patients, provided facilities, carried out the immunohistochemistry studies etc.), MP, AR, CZ, LS, CL, LA, PP, LP, AP, FG, AT, AB, RV; Development of methodology (e.g., statistical analysis, biostatistics, computational analysis) MP; CL and MC; Administrative, technical, or material support (i.e., reporting or organizing data, constructing databases): MP, EM, LA, PP, LP, AP, FG, AT, AB, RV, CL, AR; Wrote the manuscript MP; AR and VC; All authors read and approved the final manuscript.

## Supplementary Material

Additional file 1: Table S1 SMARCB1/INI1 expression profiles and molecular markers of tumor differentiation stratified according to the MMR status. **Table S2.** List of the 45 genes whose expression changes significantly correlate with *INI1* deregulation (SMARCB1 in the list) relative to Cohort I that comprises 226 patients. The negative value indicates under-expressed genes. **Figure S1.** SMARCB1/INI1 expression and *TP53* mutation status correlate with prognosis in a CRC independent data set, cohort II. **Figure S2.** Top gene networks identified through integrative pathways analysis. **Figure S3.** The gene signature mapping to chromosome 22 close to *SMARCB1/INI1* is maintained in a panel of CRC cell lines. Click here for file
