# Supplementary material for: The chromatin remodelling component SMARCB1/INI1 influences the metastatic behavior of colorectal cancer through a gene signature mapping to chromosome 22
Source: J Transl Med. 2013 Nov 28;11:297. doi: 10.1186/1479-5876-11-297 (PMC4220786; doi:10.1186/1479-5876-11-297)
Supplement: Additional file 1: Table S1 — SMARCB1/INI1 expression profiles and molecular markers of tumor differentiation stratified according to the MMR status. Table S2. List of the 45 genes whose expression changes significantly correlate with INI1 deregulation (SMARCB1 in the list) relative to Cohort I that comprises 226 patients. The negative value indicates under-expressed genes. Figure S1. SMARCB1/INI1 expression and TP53 mutation status correlate with prognosis in a CRC independent data set, cohort II. Figure S2. Top gene networks identified through integrative pathways analysis. Figure S3. The gene signature mapping to chromosome 22 close to SMARCB1/INI1 is maintained in a panel of CRC cell lines. [file 1479-5876-11-297-S1.doc]

**Additional file 1**

**Table S1**

|  | |  | **MMR status** | |  |
| --- | --- | --- | --- | --- | --- |
| **Parameters** | | **n** | **MMR + (%) MMR - (%)** | | ***p* value** |
| Sex | F | 49 | 34 (69.4) | 15 (30.6) | 0.09 |
|  | M | 85 | 69 (81.2) | 16 (18.8) |  |
| Loc | Proximal | 51 | 32 (62.7) | 19 (37.3) | 0.002* |
|  | Distal | 83 | 71 (85.5) | 12 (14.5) |  |
| Grade | Well/mod | 109 | 90 (82.6) | 19 (17.4) | 0.001* |
|  | poor | 25 | 13 (52) | 12 (48) |  |
| CDX2 | Neg | 15 | 3 (20) | 12 (80) | 0.0001* |
|  | Pos | 119 | 100 (84) | 19 (16) |  |
| CK20 | Neg | 46 | 29 (63) | 17 (37) | 0.006* |
|  | Pos | 88 | 74 (84) | 14 (16) |  |
| Vim | Neg | 111 | 85 (76.6) | 26 (23.4) | 0.010* |
|  | Low | 17 | 16 (94) | 1 (6) |  |
|  | High | 6 | 2 (33.3) | 4 (66.7) |  |
| p53 | Neg | 39 | 24 (61.5) | 15 (38.5) | 0.001* |
|  | Low | 46 | 33 (71.7) | 13 (28.3) |  |
|  | High | 49 | 46 (93.9) | 3 (6.1) |  |
| INI1 | Neg | 15 | 9 (60) | 6 (40) | 0.221 |
|  | Low | 75 | 58 (77.3) | 17 (22.7) |  |
|  | High | 44 | 36 (81.8) | 8 (8.2) |  |
| Total |  | 134 | 103(77) | 31 (23) |  |

**Table S1. SMARCB1/INI1 expression profiles and molecular markers of tumor differentiation stratified according to the MMR status. Abbreviations:** Loc, localization; Proximal: caecum, ascending and transverse colon; Distal: descending and sigmoid colon, rectum; Well/mod, well and moderately differentiated; Poor, poorly differentiated adenocarcinoma; Vim, vimentin; *Chi-square statistically significant at 0.01 level.

**Table S2**

| Probe Set ID | Gene Symbol | INI1_Down | INI1_Up | Regulation |
| --- | --- | --- | --- | --- |
| 201117_s_at | CPE | 0,37941465 | -0,5062409 | down |
| 201251_at | PKM2 | -0,29143026 | 0,309651 | up |
| 201367_s_at | ZFP36L2 | -0,38442478 | 0,37868348 | Up |
| 201755_at | MCM5 | -0,28293616 | 0,3260459 | up |
| 202315_s_at | BCR | -0,31225517 | 0,3233679 | up |
| 202407_s_at | PRPF31 | -0,33437487 | 0,25896725 | up |
| 202589_at | TYMS | -0,23102841 | 0,5018179 | up |
| 202779_s_at | UBE2S | -0,5038876 | 0,400629 | up |
| 202870_s_at | CDC20 | -0,37049514 | 0,23847267 | up |
| 203239_s_at | CNOT3 | -0,32715183 | 0,2666084 | up |
| 203751_x_at | JUND | -0,2976713 | 0,30815348 | up |
| 203755_at | BUB1B | -0,46429348 | 0,1491201 | up |
| 203967_at | CDC6 | -0,42263114 | 0,24523476 | up |
| 204126_s_at | CDC45 | -0,31079444 | 0,27842355 | up |
| 204667_at | FOXA1 | -0,33998236 | 0,34224746 | up |
| 204693_at | CDC42EP1 | -0,24633852 | 0,38585418 | up |
| 204875_s_at | GMDS | -0,33664924 | 0,35895064 | up |
| 205164_at | GCAT | -0,24823813 | 0,35384786 | up |
| 206445_s_at | PRMT1 | -0,32375267 | 0,2746751 | up |
| 208817_at | COMT | -0,32352707 | 0,30626214 | up |
| 209894_at | LEPR | 0,22824799 | -0,36888352 | down |
| 212016_s_at | PTBP1 | -0,3276136 | 0,26125795 | up |
| 212167_s_at | SMARCB1 | -0,31861517 | 0,30159876 | up |
| 212218_s_at | FASN | -0,29992402 | 0,3209584 | up |
| 213041_s_at | ATP5D | -0,25388658 | 0,41272363 | up |
| 213599_at | OIP5 | -0,34996808 | 0,26342565 | up |
| 213606_s_at | ARHGDIA | -0,49918354 | 0,13411039 | up |
| 216237_s_at | MCM5 | -0,34651124 | 0,27571294 | up |
| 217294_s_at | ENO1 | -0,47216675 | 0,18265325 | up |
| 217871_s_at | MIF | -0,36523482 | 0,23895529 | up |
| 217996_at | PHLDA1 | -0,28259638 | 0,39622697 | up |
| 218051_s_at | NT5DC2 | -0,37034833 | 0,31705996 | up |
| 221577_x_at | GDF15 | -0,41330102 | 0,30497354 | up |
| 222037_at | MCM4 | -0,459969 | 0,18010621 | up |
| 87100_at | ABHD2 | -0,48052695 | 0,20711039 | up |
| 222501_s_at | REPIN1 | -0,21467103 | 0,4114809 | up |
| 223395_at | ABI3BP | 0,2505283 | -0,39655095 | down |
| 224982_at | AKT1S1 | -0,35702246 | 0,29006675 | up |
| 225767_at | LOC284801 | -0,65207124 | 0,21648628 | up |
| 227134_at | SYTL1 | -0,15430307 | 0,4814137 | up |
| 228582_x_at | MALAT1 | 0,059804603 | -0,6605266 | down |
| 1554408_a_at | TK1 | -0,3322812 | 0,25479108 | up |
| 1554696_s_at | TYMS | -0,2138061 | 0,54816836 | up |
| 1556834_at | LOC100652770 | 0,12983163 | 0,7320748 | up |
| 1564494_s_at | P4HB | -0,40463936 | 0,21337932 | up |
| 1565483_at | EGFR | 0,17507568 | -0,41920903 | down |

**Table S2.** List of the 45 genes whose expression changes significantly correlate with *INI1* deregulation (SMARCB1 in the list) relative to Cohort I that comprises 226 patients. The negative value indicates under-expressed genes.

**Supplementary Figure**

**Figure S1. SMARCB1/INI1 expression and *TP53* mutation status correlate with prognosis in a CRC independent data set, cohort II.**

1. Kaplan-Meier analysis is carried out taking into account SMARCB1/INI1-negative tumors (down-regulated) and the *TP53* mutation status. Wild type (WT) or mutated (Mut) groups are compared to all others. **(B)** The survival curve shows that *SMARCB1/INI1* differential expression is correlated with patients’ prognosis. **(C)** The most varied top genes, that show down- or up-regulation for *SMARCB1/INI1* across tumor samples, are localized on [chromosome 22](http://ghr.nlm.nih.gov/chromosome/22). The *p* values are reported in each graph.

**Figure S2. Top gene networks identified through integrative pathways analysis.**

**(A)** The differentially expressed genes, including *SMARCB1/INI1,* are enriched in GO biological processes. **(B)** Top gene networks are identified through integrative pathways analysis centered on *SMARCB1/INI1* gene hub. The up- (color) or down-regulated genes in tumors (white) are indicated.

**Figure S3. The gene signature mapping to chromosome 22 close to *SMARCB1/INI1* is maintained in a panel of CRC cell lines**

**(A)** Western blot analysis of SMARCB1/INI1 in a panel of CRC derived cell lines. **(B)** The histogram reports quantitative expression levels of SMARCB1/INI1 after normalization to β-actin. **(C)** Migration potential of the indicated CRC cell lines is assessed by the wound-healing assay; the percentage of wound closure is measured at 24 h. **(D)** qRT-PCR analysis of the indicated genes in the four CRC cell lines. Color map shows the relative gene expression as “fold-change”, normalized to *GAPDH* mRNA as calibrator. The degree of red and green colors indicates increased or decreased expression as compared to the median value set at 0.
